# Voltage-dependent G-protein regulation of Ca_V_2.2 (N-type) channels

**DOI:** 10.1126/sciadv.adp6665

**Published:** 2024-09-11

**Authors:** Michelle Nilsson, Kaiqian Wang, Teresa Mínguez-Viñas, Marina Angelini, Stina Berglund, Riccardo Olcese, Antonios Pantazis

**Affiliations:** ^1^ Division of Cell and Neurobiology, Department of Biomedical and Clinical Sciences, Linköping University, SE-581 85 Linköping, Sweden.; ^2^ Department of Anesthesiology and Perioperative Medicine, David Geffen School of Medicine, University of California, Los Angeles, Los Angeles, CA 90095, USA.; ^3^ Department of Physiology, David Geffen School of Medicine, University of California, Los Angeles, Los Angeles, CA 90095, USA.; ^4^ Wallenberg Center for Molecular Medicine, Linköping University, SE-581 85 Linköping, Sweden.

## Abstract

How G proteins inhibit N-type, voltage-gated, calcium-selective channels (Ca_V_2.2) during presynaptic inhibition is a decades-old question. G proteins Gβγ bind to intracellular Ca_V_2.2 regions, but the inhibition is voltage dependent. Using the hybrid electrophysiological and optical approach voltage-clamp fluorometry, we show that Gβγ acts by selectively inhibiting a subset of the four different Ca_V_2.2 voltage-sensor domains (VSDs I to IV). During regular “willing” gating, VSD-I and -IV activations resemble pore opening, VSD III activation is hyperpolarized, and VSD II appears unresponsive to depolarization. In the presence of Gβγ, Ca_V_2.2 gating is “reluctant”: pore opening and VSD I activation are strongly and proportionally inhibited, VSD IV is modestly inhibited, while VSD III is not. We propose that Gβγ inhibition of VSDs I and IV underlies reluctant Ca_V_2.2 gating and subsequent presynaptic inhibition.

## Teaser

The voltage sensors of a channel that controls synaptic transmission respond differentially to electrical and biochemical signals.

## INTRODUCTION

**Fig. 1.**
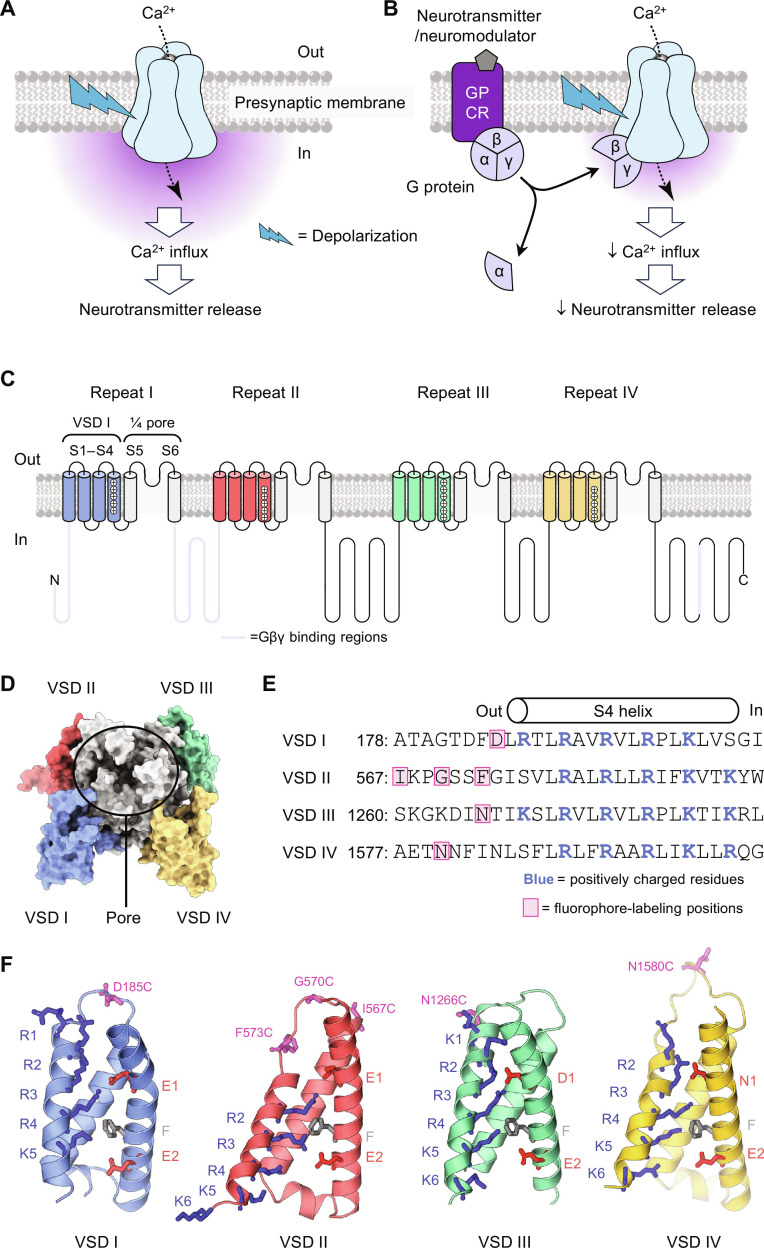
Ca_V_2.2 function, G-protein inhibition, and structure. (**A**) The arrival of an action potential to the presynaptic terminus triggers the activation of Ca_V_2.2 channels, Ca^2+^ influx, and neurotransmitter release (*1*). (**B**) Neurotransmitters and neuromodulators can activate their cognate, presynaptic G-protein–coupled receptors (GPCRs) triggering G-protein hydrolysis and release. The Gβγ complex directly inhibits the voltage-dependent opening of Ca_V_2.2, reducing Ca^2+^ influx and neurotransmitter release (*1*). (**C**) Membrane topology of the Ca_V_2.2 pore-forming subunit (α_1B_). It comprises four homologous repeats, each spanning the membrane six times. The first four transmembrane segments of each repeat (S1 to S4) assemble into a distinct voltage-sensor domain (VSD), while S5 and S6 from all repeats form the central pore. Gβγ is thought to bind to cytosolic loop regions (magenta): the N terminus, the repeat I-II loop, and the C terminus. (**D**) Structure of a human Ca_V_2.2 channel (*6*). (**E**) Amino acid sequence diversity among the four Ca_V_2.2 repeats. Positively charged residues (blue) drive VSD activation upon membrane depolarization. Pink boxes show cysteine-substituted positions for fluorescence labeling and voltage-clamp fluorometry. (**F**) Ribbon structures of VSDs I to IV (*6*). Pink residues show cysteine-substituted positions used for site-directed fluorescent labeling. Cobalt-blue residues are positively charged arginines (R) and lysines (K). Gray residues are phenylalanines (F) indicating the charge transfer center. Red residues are negatively charged countercharges (CC) in S2. Note the differences in gating charges R1-K6 and that VSD II was resolved in a down state (resting, gating charges below F).

N-type voltage-gated calcium channels Ca_V_2.2 are found at the presynaptic terminal of neurons in the central and peripheral nervous systems, where their activation initiates calcium-dependent neurotransmitter release (Fig. 1A) (*1*). Ca_V_2.2 channels are renowned for their abundance in nociceptors and their role in the development and treatment of chronic pain (*1*). Neurotransmitters and neuromodulators such as noradrenalin, serotonin, γ-aminobutyric acid (*2*), and opioids (*3*) can inhibit Ca_V_2.2. This was initially demonstrated in 1978 in dorsal root ganglion (DRG) neurons (*2*) and was eventually ascribed to G-protein–induced inhibition of Ca_V_2.2 (*1*, *4*). The Gβγ complex directly inhibits Ca_V_2.2 opening, reducing calcium influx and subsequent neurotransmitter release (Fig. 1B) (*1*).

The Ca_V_2.2 α_1B_ pore-forming subunit consists of four interlinked repeats (I to IV) that form a central calcium-conducting pore domain (transmembrane segments S5 and S6 from each repeat) and four voltage-sensor domains (VSDs; segments S1 to S4 from each repeat) that surround and control the opening of the pore (Fig. 1, C and D) (*5*–*7*). The four VSDs differ in amino acid composition and likely respond differentially to depolarization (*8*, *9*). Binding sites for Gβγ have been identified in the N terminus, repeat I-II loop, and C terminus of Ca_V_2.2 (Fig. 1C) (*10*–*12*). Curiously, G-protein inhibition is voltage dependent. As described by Bean (*4*), G-protein inhibition changes calcium-channel gating from willing to reluctant, shifting voltage-dependent activation toward depolarized potentials. G proteins can also inhibit Ca_V_2.2 gating currents (*13*–*15*). Collectively, this points to an important role of the VSDs. Here, we present voltage-clamp fluorometry (VCF) data illuminating the activation of individual voltage sensors and their inhibition by G proteins—fundamental events controlling calcium-mediated transmitter release.

## RESULTS

### Ca_V_2.2 VSDs have diverse responses to depolarization

To determine the voltage-sensing properties of the Ca_V_2.2 VSDs, we implemented VCF (*16*–*18*). VCF enables the optical dissection of the individual VSDs in conducting Ca_V_2.2 macromolecular complexes (α_1B_/α_2_δ-1/β_2a_) under physiologically relevant conditions. Ca_V_2.2 channel complexes were expressed in *Xenopus laevis* oocytes. MTS-TAMRA [MTS-5(6)-carboxytetramethylrhodamine], an environment-sensitive fluorophore, was conjugated to a cysteine substituted at the S3-S4 linker of each VSD (Fig. 1E), reporting VSD activation as fluorescence deflections (Δ*F*). The membrane potential was controlled with the cut-open oocyte Vaseline gap (COVG) method (*19*–*21*).

**Fig. 2.**
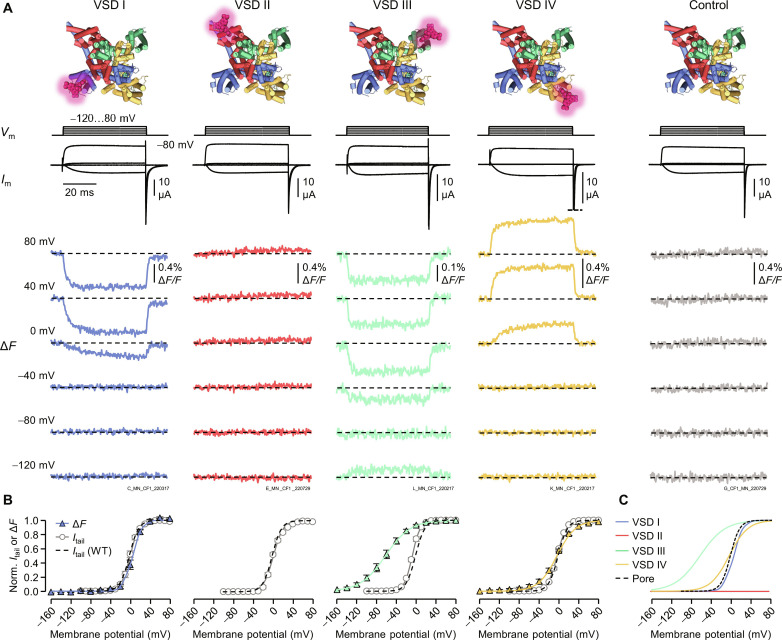
Distinct voltage-sensor activation properties of the Ca_V_2.2 VSDs. (**A**) Voltage-clamp fluorometry recordings in Ca_V_2.2. Membrane potential (*V*_m_) steps, corresponding current (*I*_m_) traces, and simultaneously acquired fluorescence deflections (Δ*F*) from each VSD, as well as control channels without substituted Cys. Note that VSD II in this and other experimental conditions (figs. S1 and S2) showed no evidence of voltage-dependent activation. (**B**) Normalized tail current–voltage (*I*_tail_-*V*) and Δ*F*-voltage (Δ*F*-*V*) curves showing the voltage-dependent pore opening and VSD activation, respectively. The gray dotted line shows the wild-type (control) *I*_tail_-*V* curve for comparison (*V*_0.5_ = 1.0 ± 1.7 mV, *z* = 2.9 ± 0.2 *e*_0_, *n* = 6). Boltzmann distribution parameters: VSD I *V*_0.5_ = 4.7 ± 2.6 mV, *z* = 3.0 ± 0.3 *e*_0_, *n* = 5. VSD III *V*_0.5_ = −62.8 ± 5.6 mV, 1.1 ± 0.1 *e*_0_, *n* = 7. VSD IV *V*_0.5_ = −5.9 ± 1.5 mV, *z* = 1.6 ± 0.2 *e*_0_, *n* = 12. Error bars represent SEM. (**C**) The voltage-dependence curves from (B) shown together for comparison and to accentuate the functional diversity of the four Ca_V_2.2 VSDs. As VSD II is apparently voltage insensitive, its voltage dependence is represented by a flat red line.

Ca_V_2.2 cysteine variants of VSDs I (D185C), III (N1266C), and IV (N1580C) generated reliable Δ*F* upon voltage-dependent activation (Fig. 2A) while maintaining wild-type–like pore-opening voltage dependence (Fig. 2B). Δ*F* from VSDs I and III was quenched upon depolarization (downward deflections), while Δ*F* from VSD IV was unquenched (upward deflection) (Fig. 2A). All VSDs responded differently to voltage (Fig. 2), as suggested by their distinct amino acid composition and structural poses (Fig. 1, E and F) (*6*, *7*). VSD II did not generate any Δ*F*, comparable to channels without substituted cysteines, regardless of the positions tested (Fig. 2A and fig. S1). This suggests that VSD II does not respond to depolarization. This is a remarkable finding that explains why recent structures of Ca_V_2.2, acquired in the absence of an electric field (0 mV), revealed S4_II_ in a resting conformation (Fig. 1F) (*6*, *7*).

VSD I activation and pore opening appeared to be coupled, as observed by the closely overlapping fluorescence deflection–voltage (Δ*F*-*V*, blue) and tail current–voltage (*I*_tail_-*V*, black) curves (Fig. 2, B and C). Specifically, VSD I activated with a half-activation potential *V*_0.5_ = 4.7 ± 2.6 mV and had an apparent voltage sensitivity *z* = 3.0 ± 0.33 *e*_0_ (*n* = 5). This was comparable to the pore opening of this construct (*V*_0.5_ = 0.091 ± 2.6 mV, *z* = 2.8 ± 0.19 *e*_0_, *n* = 5).

VSD II did not respond to voltage changes. We tested three, uniformly spaced, labeling positions in the S3_II_-S4_II_ linker: I567C, G570C, and F573C (Fig. 1E). Despite high functional expression, no Δ*F* could be observed, even at extreme voltages (−160 to 160 mV; Fig. 2A and fig. S1). To prevent any occurrence of stable quenching, we removed a nearby tryptophan (W564F), as in a previous VCF study on BK channels (*22*), but this made no difference (fig. S1). We also tested labeling with fluorophores with a longer stalk, 6-TAMRA C6 maleimide (*23*) or Alexa Fluor 488 C_5_ maleimide, but this did not generate any Δ*F* (fig. S1). Initial structures of Ca_V_2.2 hinted that S4_II_ might be stabilized in the down state by a PIP_2_ molecule (*6*, *7*), and PIP_2_ is known to modulate Ca_V_2.2 (*24*). Thus, we depleted PIP_2_ using the voltage-sensitive phosphatase DrVSP, as previously described (*25*). PIP_2_ depletion was confirmed as DrVSP-expressing cells had a significant reduction in *I*_tail_ after a depolarizing pulse, compared to cells that did not express DrVSP; still, we did not observe Δ*F* (fig. S2). Subsequent structures of the Ca_V_2.3 channel also showed S4_II_ in a down state, in the absence of PIP_2_ (*26*, *27*), suggesting that the “locked down” S4_II_ is a feature of the Ca_V_2 family independently of PIP_2_.

VSD III activated at notably negative potentials and had a half-activation potential of −62.8 ± 5.6 mV, and low apparent voltage sensitivity at 1.1 ± 0.1 *e*_0_ (*n* = 7) (Fig. 2, B and C). Hence, ~50% of Ca_V_2.2–VSD III would be active at resting membrane potentials. At −20 mV, 4% of channels were open, while 90% of VSD III were activated. Last, VSD IV had a half-activation potential of −5.9 ± 1.5 mV, close to that of pore opening, and a low apparent voltage sensitivity of 1.6 ± 0.2 *e*_0_ (*n* = 12) (Fig. 2, B and C).

### VSD I responds more strongly than VSDs III and IV to pre-pulse facilitation

**Fig. 3.**
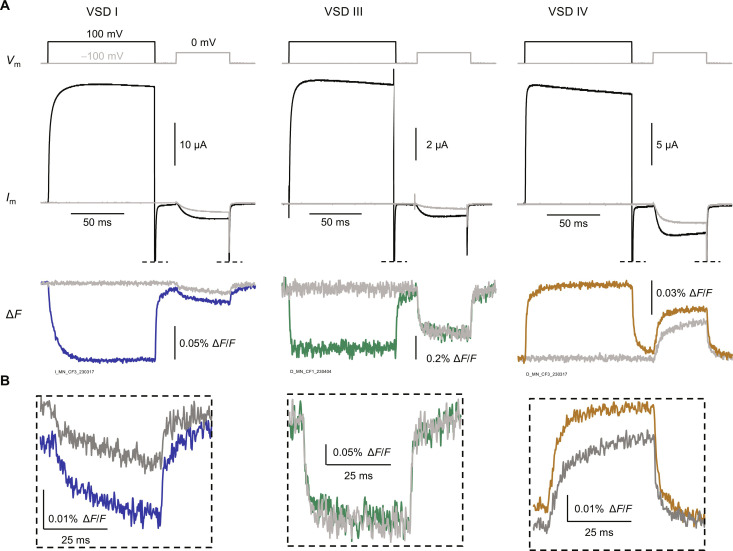
A distinct voltage sensor facilitates pre-pulse facilitation in the presence of Gβγ. (**A**) Exemplary recordings of VSD pre-pulse facilitation in cells expressing α_1B_/α_2_δ-1/β_2a_/Gβ1/Gγ2. Top: Steps of membrane potential (*V*_m_) (i) without pre-pulse (gray) and (ii) with 100-ms facilitating pre-pulse at 100 mV (black), both followed by a test pulse at 0 mV. Middle: Corresponding facilitating barium current (*I*_Ba_). Bottom: Fluorescence deflections (Δ*F*) from VSD I, III, and IV without (gray) and with (blue, green, and yellow, respectively) facilitating pre-pulse. (**B**) Close-up of VSDs I, III, and IV Δ*F* during the 0-mV test pulse.

With the additional dimension of individual voltage-sensor resolution now available from VCF, we explored the mechanistic details of G-protein inhibition of Ca_V_2.2. To recapitulate Ca_V_2.2 inhibition by G proteins in our experimental paradigm, we coexpressed the Ca_V_2.2 complex (α_1B_/α_2_δ-1/β_2a_) together with the human Gβ1 and Gγ2 subunits. A hallmark of Gβγ inhibition is “pre-pulse facilitation” (*28*, *29*). This describes the current increase observed following a strongly depolarizing pre-pulse during Gβγ inhibition and is thought to be mediated by the transient unbinding of Gβγ and relief of Gβγ inhibition (*29*). When we optically tracked Ca_V_2.2 VSD activation in the presence of Gβγ, we found that the VSDs facilitated to a different extent (Fig. 3): VSD I had the most prominent facilitation, VSD III did not respond to pre-pulse facilitation, and VSD IV had an intermediate response. This revealed that G-protein–coupled receptor (GPCR) signaling modulates the Ca_V_2.2 VSDs, in a VSD-selective manner.

### Gβγ makes VSDs I and IV “reluctant” to activate

**Fig. 4.**
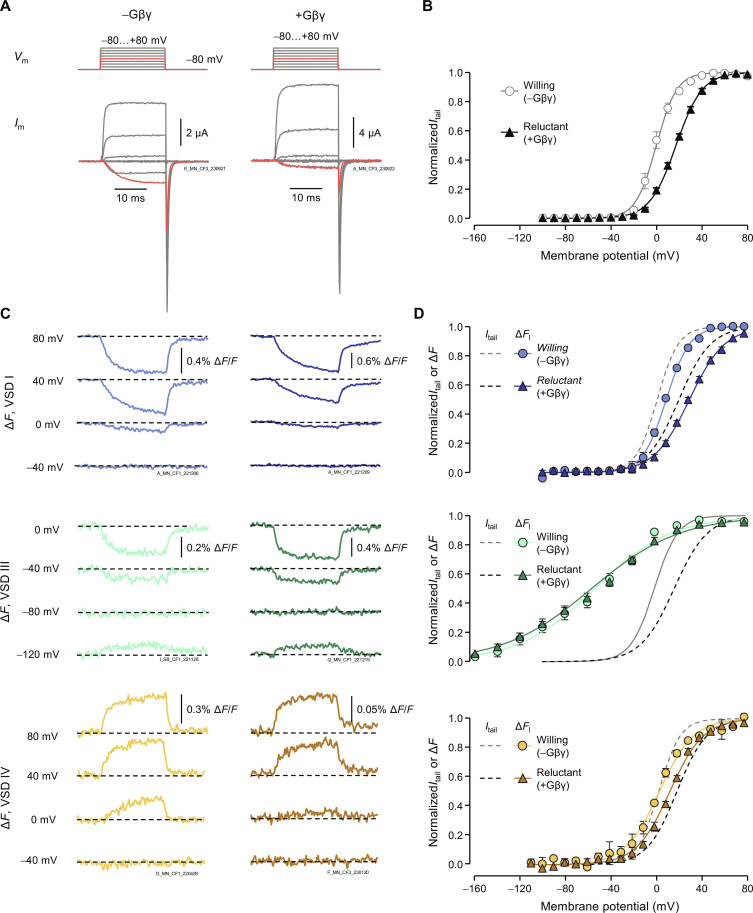
G-protein–induced inhibition of VSDs I and IV. (**A**) Steps of membrane potential (*V*_m_) and corresponding current traces without (−Gβγ, “willing”) or with Gβ1 and Gγ2 (+Gβγ, “reluctant”) from channels labeled in VSD I. (**B**) Normalized (*I*_tail_-*V*) curves showing the voltage-dependent opening of wild-type Ca_V_2.2: −Gβγ: *V*_0.5_ = 3.3 ± 0.9 mV, *z* = 3.2 ± 0.2, *n* = 9; +Gβγ: *V*_0.5_ = 17 ± 0.8 mV, *z* = 2.3 ± 0.1 *e*_0_, *n* = 7. (**C**) Representative fluorescence deflections (Δ*F*) from VSDs I, III, and IV in the presence or absence of Gβγ. (**D**) Normalized Δ*F*-voltage (Δ*F*-*V*) curves showing voltage-dependent activation of VSD I, III, and IV. VSD I: −Gβγ: *V*_0.5_ = 10 ± 1.3 mV, *z* = 2.5 ± 0.2 *e*_0_, *n* = 5; +Gβγ: *V*_0.5_ = 31 ± 1 mV, *z* = 1.7 ± 0.1 *e*_0_, *n* = 7. VSD III: −Gβγ: *V*_0.5_ = −54 ± 7 mV, *z* = 0.8 ± 0.1 *e*_0_, *n* = 6; +Gβγ: *V*_0.5_ = −54 ± 5 mV, *z* = 0.7 ± 0.03 *e*_0_, *n* = 9. VSD IV: −Gβγ: *V*_0.5_ = 1.5 ± 1.6 mV, *z* = 2.1 ± 0.3 *e*_0_, *n* = 10; +Gβγ: *V*_0.5_ = 14 ± 1 mV, *z* = 1.9 ± 0.2 *e*_0_, *n* = 10. The black solid line (−Gβγ) and black dotted line (+Gβγ) show *I*_tail_-*V* curves from the corresponding cysteine variant that Δ*F* was collected from. Error bars are SEM.

To further determine Gβγ inhibition of the VSDs, we studied their voltage-dependent activation in the absence or presence of Gβγ. To prevent the relief of Gβγ inhibition (dependent on time and voltage), we used shorter (20 ms) activation pulses. As expected, Gβγ shifted the *V*_0.5_ of Ca_V_2.2 pore opening toward positive potentials, by 14 ± 0.8 mV (*P* = 3 × 10^−7^; Fig. 4, A and B).

We found that VSD I activation is strongly inhibited by Gβγ. Gβγ shifted the VSD I *V*_0.5_ by 21 ± 1.0 mV, *P* = 8 × 10^−8^, similar to channel opening in the same cells (Fig. 4, C and D). To evaluate whether the effects on VSD I activation were mediated via the canonical Gβγ binding sites, we introduced point mutation R54A, previously shown to abolish G-protein inhibition (*30*). Gβγ inhibition of both the pore opening and the VSD I activation was eliminated (fig. S3). The R54A mutation appeared to produce a depolarizing shift in the voltage dependence of both VSD I activation and channel opening. A plausible interpretation of this effect is that R54 electrostatically opposes VSD I deactivation, effectively facilitating VSD I activation and, consequently, channel opening. In contrast to VSD I, VSD III was not modulated by Gβγ, Δ*V*_0.5_ = −0.1 ± 5.3 mV, *P* = 0.99 (Fig. 4, C and D)*.* VSD IV displayed modest inhibition by Gβγ compared to VSD I, Δ*V*_0.5_ = 13 ± 1 mV, *P* = 1 × 10^−6^ (Fig. 4, C and D).

### VSD I activation and pore opening are strongly and proportionately inhibited by Gβγ during DRG action potentials

**Fig. 5.**
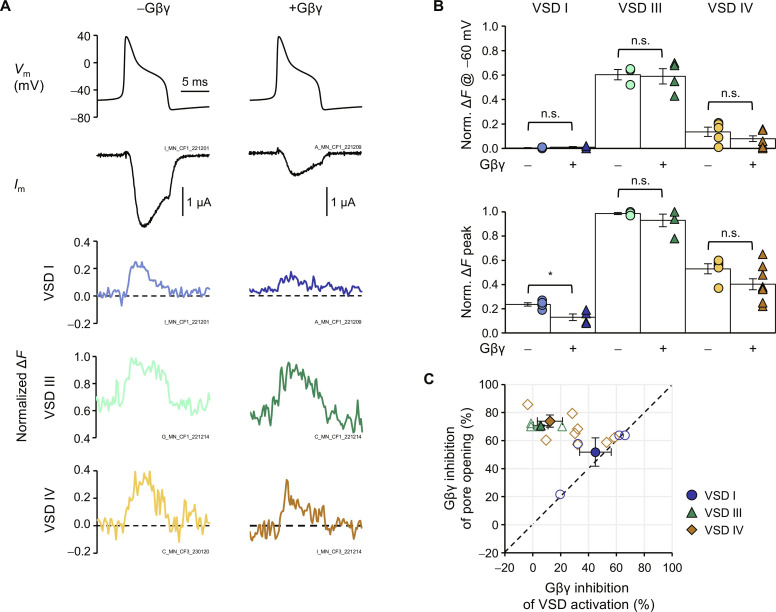
VSD I activation and pore opening are strongly and proportionally inhibited by Gβγ during a DRG action potential. (**A**) Top: Action potential clamp (*V*_m_) in cells expressing α_1B_/α_2_δ-1/β_2a_ without (−Gβγ) or with Gβ1 and Gγ2 (+Gβγ). Middle: Corresponding current traces. Bottom: Normalized Δ*F* from VSDs I, III, and IV. (**B**) Top: Normalized Δ*F* at resting membrane potential (*V*_rest_, −60 mV). VSD I: −Gβγ: 0.0 ± 0.0%, *n* = 5; +Gβγ: 0.0 ± 0.0%, *n* = 4. VSD III: −Gβγ: 60 ± 4%, *n* = 3; +Gβγ: 59 ± 6%, *n* = 4, *P* = 0.88. VSD IV: −Gβγ: 14 ± 4%, *n* = 5; +Gβγ: 8 ± 2%, *n* = 9, *P* = 0.21. Bottom: Normalized peak Δ*F* VSD I: −Gβγ: 24 ± 1%, *n* = 5; +Gβγ: 13 ± 3%, *n* = 4, *P* = 0.007. VSD III: −Gβγ: 99 ± 1%, *n* = 3; +Gβγ: 93 ± 5%, *n* = 4, *P* = 0.4. VSD IV: −Gβγ: 53 ± 4%, *n* = 5; +Gβγ: 40 ± 5%, *n* = 9, *P* = 0.09. Tests were two-tailed, unpaired *t* tests. n.s., nonsignificant. *P < 0.05. (**C**) Gβγ inhibition of pore opening (conductance) plotted against Gβγ inhibition of VSD activation. Open symbols represent individual experiments, and filled symbols are means. The dashed line represents 1:1 inhibition between the pore opening and the voltage sensors. VSD I inhibition tracked conductance inhibition (*P* = 1.0). On the other hand, inhibition of VSDs III and IV did not correspond to conductance inhibition (*P* = 0.011 and 4.9 × 10^−4^, respectively). Tests were two-sample Kolmogorov-Smirnov type. Error bars are SEM.

Ca_V_2.2 is the predominant synaptic calcium channel in nociceptors (*31*–*34*) and is important in pain sensitivity and the development of chronic pain (*35*–*37*). To investigate VSD operation under a physiological stimulus, we implemented an action potential clamp with a waveform characteristic of small, unmyelinated nociceptive DRG neurons (*34*, *38*, *39*). At the resting membrane potential (*V*_rest_, −60 mV) channels were closed (Fig. 5, A and B). In the case of VSD I–labeled channels, the peak macroscopic conductance was observed during the plateau phase of the action potential, reaching 17 ± 1% of the maximum, as normalized by a standard “*I*_tail_-*V*” protocol on the same cell. In the presence of Gβγ, the peak conductance was significantly reduced by approximately half, to 8 ± 2% (*P* = 0.004; Fig. 5C).

At *V*_rest_, VSD I was not activated, while already 60 ± 4% of VSD III and 14 ± 4% of VSD IV were active (Fig. 5, A and B)—assuming that limiting VSD activation was 100% and the fluorescence signal tracked VSD activation in an all-or-nothing manner. These fractions did not significantly change in the presence of Gβγ (VSD I: *P* = 0.33; VSD III: *P* = 0.88; VSD IV: *P* = 0.21).

Peak VSD activation was defined as the maximum amplitude of the Δ*F*, normalized by a standard “Δ*F*-*V*” protocol. Similar to pore opening, VSD I reached 24 ± 1% activation and was significantly reduced to 13 ± 3% in the presence of Gβγ (*P* = 0.007; Fig. 5). VSD III reached maximal activation independently of Gβγ (−Gβγ; 98 ± 10%; +Gβγ: 94 ± 5%; *P* = 0.4). Last, 53 ± 4% of VSD IV activated at peak, which was not significantly different in the presence of Gβγ (40 ± 5%; *P* = 0.09). To better illustrate the timing of VSD activations and pore opening, the activity traces were used to annotate the Ca_V_2.2 structure in movie S1.

## DISCUSSION

Our most recent knowledge on the VSDs of Ca_V_2.2 came from the atomic structures resolved by cryogenic electron microscopy, effectively at 0 mV (*6*, *7*). However, the dynamic responses of these structures to electrical signals and their regulation by G proteins were unknown. Our optical investigation of the Ca_V_2.2 VSDs under physiologically relevant conditions revealed that these domains exhibit distinct voltage-sensing properties and regulation by G proteins. In summary, we found that (i) VSDs I, III, and IV activate with distinct voltage dependencies, likely contributing in different ways to channel opening; (ii) in our tested conditions, VSD II does not respond to changes in the membrane potential; and (iii) Gβγ sets VSDs I and IV in a reluctant mode of activation.

### The Ca_V_2.2 voltage–sensing apparatus

VSD I activation and pore opening exhibit very similar voltage dependence (Figs. 2, 4, and 5), suggesting that this domain is strongly coupled to pore opening. In favor of this interpretation, action potential clamp protocols revealed that VSD I activation and pore opening are coupled in both time and voltage (Fig. 5 and movie S1). Both achieved maximal activation or open probability of roughly 20% during an action potential. Introducing a negative charge in Ca_V_2.2 S3_I_, which might inhibit VSD I activation, shifted *V*_0.5_ of pore opening by more than +10 mV (*40*).

VSD II may not be pertinent to channel opening, as discussed below. VSDs III and IV both activate within physiologically relevant potentials. VSD III, in particular, appears to be tuned to sensing the resting membrane potential. Voltage-clamp recordings and stimulation with an action potential waveform showed that 60% of VSD III is already active at resting membrane potential when channels are closed (Figs. 2, 4, and 5). This anticipates that Ca_V_2.2 channels have at least two closed states: one with VSD III in the resting conformation and another with VSD III in the active conformation. This suggests that VSD III activation does not directly drive pore opening. VSD IV has a voltage dependence approaching that of pore opening, although the latter saturates at more negative potentials than VSD IV activation (Fig. 2). One interpretation is that VSDs I, III, and IV are all required for opening of the pore, where VSD III activation is the first VSD to transition to an active state and VSDs I and/or IV are limiting factors for pore opening.

We did not observe any fluorescence deflections from VSD II despite testing multiple positions and conditions, even at extreme levels of depolarization, hyperpolarization, and expression (Fig. 2 and figs. S1 and S2). Our findings, together with recent structural evidence, support the role of the VSD II alternative to voltage sensing. In 2021, two independent studies both revealed Ca_V_2.2 channels with S4_II_ in a down state in the absence of an electric field (0 mV), while S4_I_, S4_III_, and S4_IV_ were in an up state (*6*, *7*). Later structures of the closely related Ca_V_2.3 channel also demonstrated resting VSD II states (*26*, *27*). Moreover, Ca_V_2.3 channels can undergo voltage-dependent opening upon neutralization of S4_II_ gating charges, with only a modest shift in *V*_0.5_ (*27*). VCF has revealed that VSD II does undergo voltage-dependent activation in Ca_V_1 channels (*8*, *9*), and accordingly, S4_II_ in resolved structures of Ca_V_1 and Ca_V_3 channels is in an active conformation (*41*–*43*). We propose that a voltage-insensitive VSD II is a signature of Ca_V_2 family channels, where it may serve as a static structural element of the channel. Perhaps under different regulatory regimes or in other splice variants, VSD II can undergo voltage-dependent activation and contribute to Ca_V_2.2 opening and regulation.

### GPCR tuning of Ca_V_2.2 voltage dependence

We found that VSD I is strongly inhibited by Gβγ, in proportion to pore-opening inhibition, while VSD IV is modestly affected and VSD III is not (Figs. 4 and 5). Accordingly, VSD I activation is also facilitated most strongly by pre-pulse facilitation, while VSD IV shows more modest facilitation and III is unaffected (Fig. 3). This hierarchy of VSD modulation by Gβγ suggests that Gβγ directly interacts with VSD I and potentially VSD IV, to prevent activation and subsequent opening. In single-channel recordings, Gβγ inhibition manifests as the emergence of low-open-probability (*P*_O_), reluctant openings and delayed high-*P*_O_ openings (*44*). Consolidating this information and our results, we propose that Gβγ acts by inhibiting VSD I–driven channel opening (delaying high-*P*_O_ events), allowing the emergence of inefficient openings by VSD IV (low-*P*_O_ events). The previously known Gβγ binding sites may position Gβγ in close proximity to VSD I (Fig. 1C) (*10*–*12*). Gβγ-mediated stabilization of the VSD I resting conformation may be due to electrostatic interactions. Introducing a negative charge in S3_I_ (G177E) to inhibit VSD I activation resulted in similar effects as G-protein inhibition: Ca_V_2.2 opening becomes intrinsically reluctant and sensitive to pre-pulse facilitation (*40*). Neutralizing the R2 gating charge of S4_I_ in the closely related Ca_V_2.1 channel also reduced G-protein inhibition (*45*). An interaction with VSDs I and IV in a down state could account for the preferential closed-state G-protein inhibition of Ca_V_2.2 (*46*, *47*).

A means to confirm these experiments would be with receptor-mediated G-protein activation, possible to implement in oocytes (*30*, *48*). In this work, we have directly expressed Gβγ subunits in oocytes together with Ca_V_2.2 to resolve the molecular perturbations associated with this modulation. While we did not rely on GPCR activation to generate Gβγ, our approach has been shown to adequately recapitulate Ca_V_ modulation relevant to GPCR activation (*30*, *48*).

Ca_V_2.2 is an attractive target for pain management as it has been implicated both in the development and treatment of chronic pain (*1*, *35*, *37*). The inhibition of VSDs I and IV poses an interesting way of regulating Ca_V_2.2 channels. For example, a drug that mimics this natural form of inhibition—a Gβγ mimetic—could be a promising next-generation analgesic, specifically targeted to inhibit VSDs I or IV. Since these domains are transmembrane, such compounds could be developed to act from the extracellular side. Tuning ion channel activation by modulating VSDs has been shown both for toxins, drug-like compounds, and endogenous molecules (*49*).

In conclusion, we show that Ca_V_2.2 VSDs respond differently to voltage and that this asymmetry extends to their modulation by other proteins. Gβγ, the result of GPCR signaling in the presynaptic terminal, specifically acts by preventing the activations of VSDs I and IV.

## MATERIALS AND METHODS

### Molecular biology

The following constructs were used: human CACNA1B [hα1B; variant +e10a, +18a, Δ19a, +e31a, +e37b, and + e46; Addgene #62574, a gift from D. Lipscombe (*50*)], rabbit CACNA2D1 (α_2_δ-1; UniProt accession no. P13806), rat CACNB2a (β_2a_; UniProt accession no. Q8VGC3), rabbit CACNB3 (β_3_; UniProt accession no. P54286), human GNB1 [Gβ1; #140987, a gift from B. Roth (*51*)], human GNG2 (Gγ2; Addgene #67018, a gift from C. Berlot), and zebrafish voltage-sensitive phosphatase [DrVSP; a gift from Y. Okamura (*52*)]. Genes in cell expression vectors were subcloned to the *Z*-vector for in vitro transcription and oocyte expression (*53*). Mutagenesis was performed using PfuUltra II Fusion High-fidelity DNA Polymerase (Agilent) or Q5 Site-Directed Mutagenesis Kit (New England Biolabs). To improve polymerase chain reaction yield with the GC-rich hα1B template, reactions were supplemented as needed with 1 to 5% dimethyl sulfoxide (DMSO; Invitrogen) and/or 1 M betaine (Thermo Fisher Scientific). Plasmid sequences were fully confirmed by sequencing. Last, cRNA was transcribed in vitro using mMESSAGE mMACHINE T7 (Thermo Fisher Scientific) or HiScribe T7 ARCA mRNA Kit (New England Biolabs), evaluated spectrophotometrically and by gel electrophoresis, and stored at −80°C.

### Oocyte preparation

Oocyte lobes were surgically removed from *X. laevis* as approved by the Linköping Animal Care and Use Committee (permit #1941), in accordance with national and international guidelines. Lobes were separated into clusters of one to five oocytes and the follicular layer was removed (i) enzymatically with Liberase (Roche) and (ii) mechanically. Both steps were done using an orbital shaker at 88 rpm at room temperature in Ca^2+^-free OR-2 solution [in mM: 82.5 NaCl, 2.5 KCl, 1 MgCl_2_, and 5 4-(2-hydroxyethyl)-1-piperazineethanesulfonic acid (HEPES) (pH 7.0)], first with Liberase for approximately 20 min, then in OR-2 for approximately 45 to 75 min. As needed, grade I defolliculated oocytes purchased from Ecocyte Bioscience were also used. Cells were stored in SOS [in mM: 100 NaCl, 2 KCl, 1.8 CaCl_2_, 1 MgCl, and 5 Hepes (pH 7.0)] at 17°C.

Stage V to VI oocytes were injected (UMP3T-1, World Precision Instruments) at the equator with 50 nl of cRNA mix containing the Ca_V_2.2 complex hα1B, α_2_δ-1, β_2a_ without or with Gβ1 and Gγ2. In PIP_2_ depletion experiments, hα1B, α_2_δ-1, β_3_, and DrVSP were co-injected. β_3_ was used as it augments Ca_V_2.2 sensitivity to PIP_2_ depletion (*25*). All subunits were injected at 0.3 μg/μl. Oocytes were incubated for 3 to 4 days at 17°C in incubation solution [in final concentration: 50% L-15 (Corning cellgro Leibovitz’s L-15), 47.5% H_2_O, 10% heat-inactivated horse serum (Gibco), penicillin/streptomycin (10^5^ U/liter and 100 mg/liter; Capricorn Scientific GmbH), amikacin (100 mg/liter; Fisher BioReagents)]. Of note, Gβγ modulation typically developed at day 4, even though Ca_V_2.2 channels were readily expressed by day 3.

### Labeling

Before VCF recordings, oocytes were labeled for 7 min on ice with 20 μM thiol-reactive fluorophore MTS-TAMRA (mixed isomers, Biotium) in a depolarizing solution [in mM: 120 K-methanesulfonate (MES), 2 Ca(MES)_2_, and 10 Hepes (pH 7.0)]. As described, in some VSD II experiments, oocytes were labeled with 10 μM 6-TAMRA C6 maleimide (AAT Bioquest) or 100 μM Alexa Fluor 488 C5 maleimide (Thermo Fisher Scientific) on ice for 30 min. All stocks were 100 mM in DMSO.

### Electrophysiology

VCF was performed at room temperature under COVG as previously described (*18*, *21*, *54*). We used a CA-1B amplifier (Dagan Corporation). All signals were sampled with a Digidata 1550B1 digitizer and pClamp 11.2.1 software (Molecular Devices). The optical setup comprised a BX51WI upright microscope (Olympus) with filters (all Semrock BrightLine: exciter: FF01-531/40-25; dichroic: FF562-Di02-25x36; emitter: FF01-593/40-25). The excitation light source was the M530L3 light-emitting diode (LED; 530 nm, 170 mW, Thorlabs) driven by a Cyclops LED driver (Open Ephys). Fluorescence emission was acquired with a LUMPLANFL 40XW water immersion objective (Olympus; numerical aperture = 0.8, working distance = 3.3 mm) and focused on a SM05PD3A Si photodiode (Thorlabs). The photocurrent was amplified with a DLPCA-200 current amplifier (FEMTO). For Alexa Fluor 488 experiments, the following filter set was used (all Semrock BrightLine): exciter: FF01-482/35-25; dichroic: FF506-DI03-25X36; emitter: FF01-524/24-25. The light source was a Thorlabs blue LED (490 nm, 205 mW, M490L4). Current and fluorescence were sampled at 25 kHz and low-pass filtered at 5 kHz.

Before mounting to the COVG chamber, each oocyte was injected with 100 nl of 100 mM BAPTA•4 K (Invitrogen), and 10 mM Hepes (pH 7.0) to prevent calcium-dependent Ca_V_2.2 regulation and oocyte currents. The external solution consisted of the following (in mM): 120 NaMES, 2 Ba(MES)_2_, and 10 Hepes (pH 7.0). The internal solution consisted of the following (in mM): 120 K-glutamate and 10 Hepes (pH 7.0). The intracellular micropipette solution consisted of the following (in mM): 2700 NaMES, 10 NaCl, and 10 Hepes (pH 7.0).

Cells were held at −80 mV and stepped to a range of voltages for 50 ms when characterizing VSDs, or 20 ms when investigating G-protein modulation. In experiments investigating G-protein modulation, a pre-pulse facilitation protocol was used to confirm Gβγ modulation (*28*, *29*). Cells were held at −100 mV and the level of facilitation was determined at a 0-mV test pulse before and after a conditioning 100-ms pre-pulse at 100 mV. In cells co-injected with Gβγ, only cells displaying ≥50% facilitation were included in the data analysis.

Action potential waveform was derived from a model based on recordings in rat DRG neurons at room temperature (*38*) in MATLAB R2022a (MathWorks) using the ode15s differential equation solver. In action potential clamp experiments, cells were held at −60 mV, as this was the cell resting potential.

### Analysis

Analysis was performed in Clampfit 11.2 (Molecular Devices), Excel (Microsoft), and MATLAB (MathWorks).

The Ca_V_2.2 tail current *I*_tail_ was obtained from the peak tail current, fitted to a Boltzmann equation

$$I_{\text{tail}}\left(V\right)=I_{\text{tail},\text{max}}/\left\{1+\text{exp}\left[zF\left(V_{0.5}-V\right)/\left(RT\right)\right]\right\}$$ (1)

where *V* is the membrane potential, *I*_tail,max_ is maximal *I*_tail_, *z* is the valence, *V*_0.5_ is half-activation potential, and *R* and *F* are the gas and Faraday constants, respectively.

Δ*F* values were obtained from the steady-state activation by the end of each pulse and fit a Boltzmann equation

$$\begin{array}{c} \Delta F\left(V\right)=\left(\Delta F_{\text{max}}-\Delta F_{\text{min}}\right)/\\ \left\{1+\text{exp}\left[zF\left(V_{0.5}-V\right)/\left(RT\right)\right]\right\}+\Delta F_{\text{min}} \end{array}$$ (2)

where Δ*F*_max_ is the maximum fluorescence deflection and Δ*F*_min_ is the minimum fluorescence deflection.

Gβγ pre-pulse facilitation “Ppf” was measured as the ratio of the current after and current before a facilitating pre-pulse, at a 0-mV test pulse at 22 ms, when facilitated current approached a plateau. This protocol was performed in all Gβγ experiments, and only cells displaying at least 50% facilitation were further analyzed

$$\text{Ppf}=\left(I_{\text{post}}/I_{\text{pre}}\right)-1$$ (3)

In Gβγ facilitation recordings of Δ*F*, facilitation was determined as above. The relative facilitation was calculated from the facilitation of the current in the same cell

$$\text{Relative facilitation}={\text{Ppf}}_{\Delta F}/{\text{Ppf}}_{I}$$ (4)

We generated a DRG neuron action potential using the Choi and Waxman formulation (*38*) and used it as a voltage command in our VCF setup.

The conductance *G* was calculated as

$$G\left(V\right)=I/\left(V-V_{\text{rev}}\right)$$ (5)

where *I* is the current across the trace, *V* is the membrane potential, and *V*_rev_ is the reversal potential as determined from an *I*_tail_-*V* 50-ms pulse protocol in the same cell obtained at the same *V*_rest_ (−60 mV). *G*(*V*) was then normalized as.

$$G_{\text{Norm}}=G\left(V\right)/\left[I_{\text{tail}\text{max}}/\left(V-V_{\text{rev}}\right)\right]$$ (6)

where *I*_tail max_ is the maximum tail current derived from the *I*_tail_-*V* protocol and *V* is the membrane potential (−60 mV).

The fluorescence was normalized by

$$F+\Delta F_{\text{min}}/-\Delta F_{\text{tot}}$$ (7)

where *F* is the fluorescence across the trace, Δ*F*_min_ is the minimum fluorescence as determined from an Δ*F*-*V* 50-ms protocol in the same cell obtained at the same *V*_rest_ (−60 mV), and Δ*F*_tot_ is the total change in fluorescence determined by the Δ*F*-*V* protocol. In fluorescence traces from VSD IV where the fluorescence deflection is positive, −Δ*F*_min_ was used. The normalized *G* or Δ*F* at *V*_rest_ was determined at −60 mV and the normalized *G* or Δ*F* peak was determined as the maximum conductance or Δ*F*.

### Statistics

All replicates (*n*-numbers) refer to experiments on different cells. All tests were two-tailed unpaired Student’s *t*-tests, except for the statistics on the relationship between VSD and conductance inhibition in action potential clamp experiments (Fig. 5C): in this case, the *p* value calculated using two-sample Kolmogorov-Smirnov tests.

### Protein structures

Ca_V_2.2 channel structures with Protein Data Bank accession number 7MIY (*6*) were rendered using The Protein Imager (*55*). Movie S1 was made on Blender 3.3.0 (The Blender Foundation).
