# Supplementary material for: Voltage-dependent G-protein regulation of CaV2.2 (N-type) channels
Source: Sci Adv. 2024 Sep 11;10(37):eadp6665. doi: 10.1126/sciadv.adp6665 (PMC11389781; doi:10.1126/sciadv.adp6665)
Supplement: Supplementary file 1 — Figs. S1 to S3 Legend for movie S1 [file sciadv.adp6665_sm.pdf]

Science Advances
AAAS

Supplementary Materials for

Voltage-dependent G protein regulation of $Ca_V2.2$ (N-type) channels

Michelle Nilsson *et al.*

Corresponding author: Antonios Pantazis, antonios.pantazis@liu.se

Sci. Adv. **10**, eadp6665 (2024)
DOI: 10.1126/sciadv.adp6665

The PDF file includes:

Figs. S1 to S3
Legend for movie S1

Other Supplementary Material for this manuscript includes the following:

Movie S1

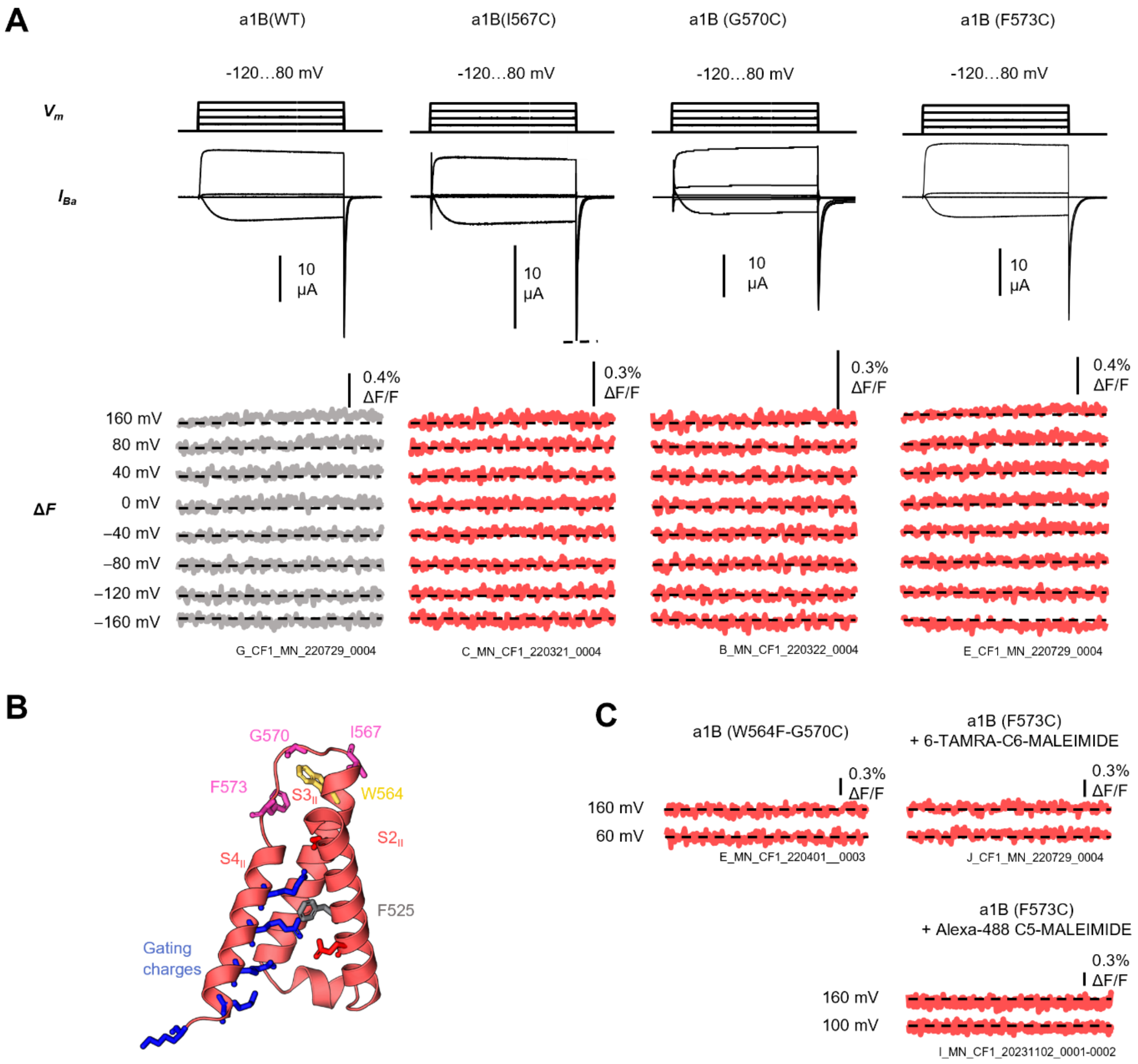

Fig. S1: VSD II is apparently voltage-independent. (A) Top: voltage-step protocol, mid: exemplary current responses (I_{Ba}), bottom: exemplary fluorescence deflections (ΔF) after staining with MTS-TAMRA. I567C, n = 1, G570C n = 5, F573C n = 6. **(B)** Resolved structure of the Ca_V2.2-VSD II (PDB ID:7MIY) (*6*). Cysteine labelling positions highlighted in pink (I567C, G570C, F573C) and removed tryptophan in yellow (W564F). **(C)** Extended labelling conditions: removal of a potential fluorophore-quenching tryptophan (W564F) (*22*, *23*) G570C, W564F n = 4, labelling F573C with 6-TAMRA-C6-MALEIMIDE (n = 3) or Alexa-488 C5-MALEIMIDE (n = 9).

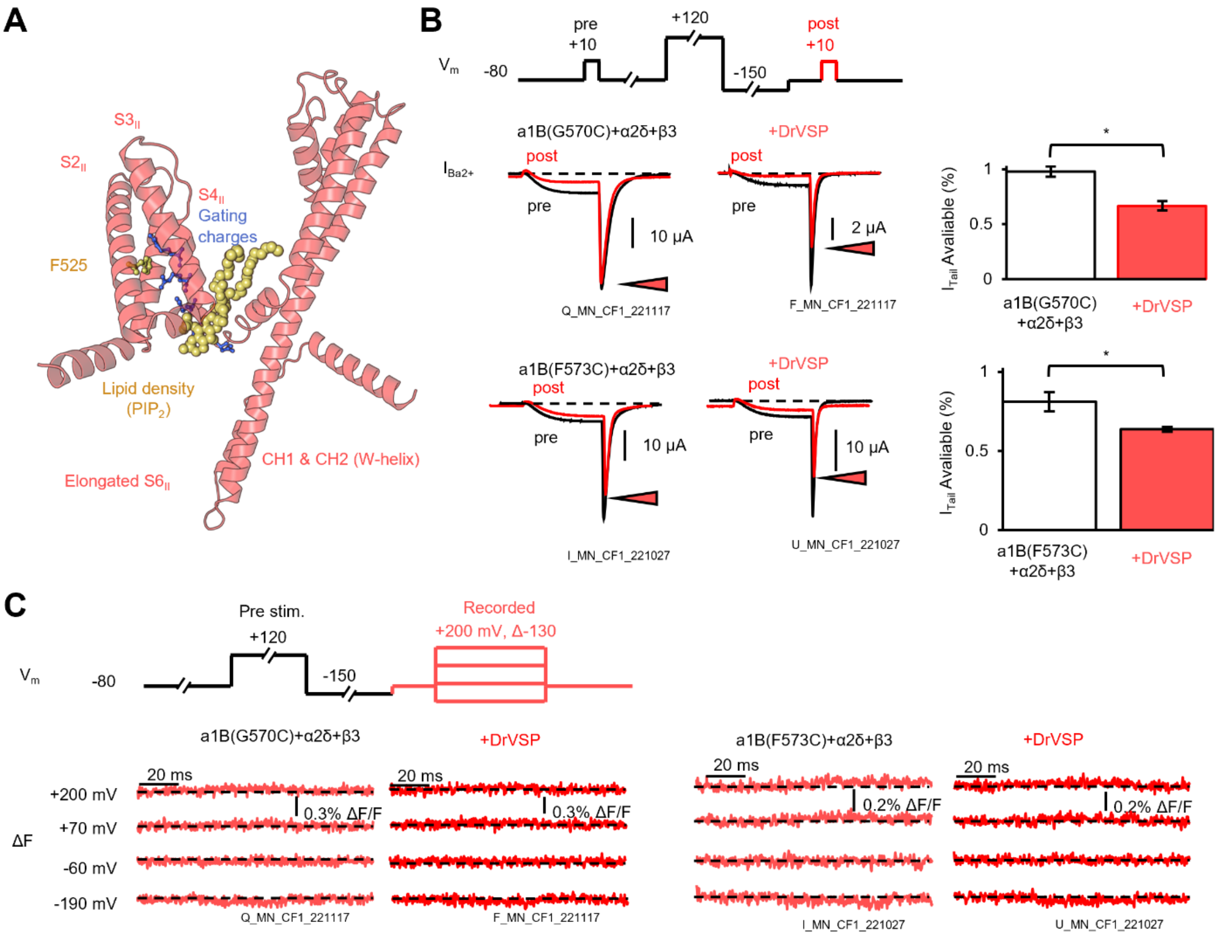

Fig. S2. PIP$_2$ depletion does not unlock VSD II activation. (A) Structure of Ca$_V$2.2 repeat II showing S4 in a down-state, potentially stabilized by a lipid density, PIP$_2$ (PDB ID:7MIY) (*6*). **(B)** Top: Depletion protocol. Pre-test pulse at 10 mV, 10 ms, DrVSP-stimulating pulse to 120 mV, 1000 ms, inactivation removal pulse to −150 mV, 400 ms, and post-test pulse at 10 mV. Bottom: available tail current before (pre) and after (post) a PIP$_2$-depleting pulse in absence or presence of DrVSP. α_{1B}(G570C)+$\alpha_2\delta$+β_3 *n* = 5, α_{1B}(G570C)+$\alpha_2\delta$+β_3+DrVSP *n* = 10, α_{1B}(F573C)+$\alpha_2\delta$+β_3 *n* = 4, α_{1B}(F573C)+$\alpha_2\delta$+β_3+DrVSP *n* = 8. **(C)**. Top: Depletion protocol followed by 50 ms test-pulses to various voltages, as indicated. Bottom: exemplary fluorescence deflections (ΔF) in absence or presence of DrVSP. α_{1B}(G570C)+$\alpha_2\delta$+β_3 *n* = 4, α_{1B}(G570C)+$\alpha_2\delta$+β_3+DrVSP *n* = 10, α_{1B}(F573C)+$\alpha_2\delta$+β_3 *n* = 3, α_{1B}(F573C)+$\alpha_2\delta$+β_3+DrVSP *n* = 8. A break (//) in the axis indicates an excluded section of timeline for clarity. Error bars are mean ± SEM, * indicates $p<0.05$.

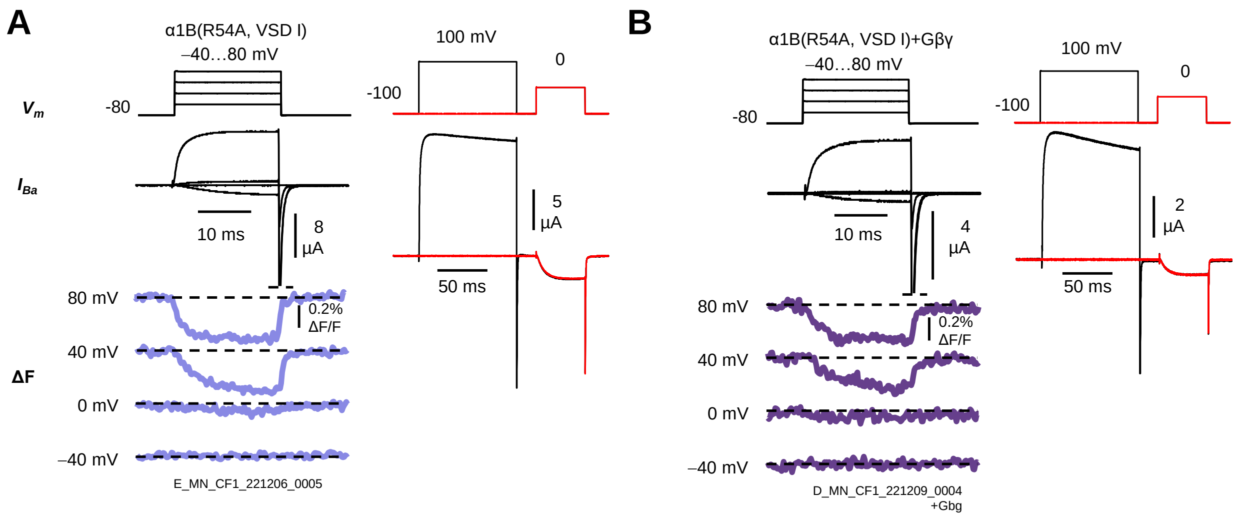

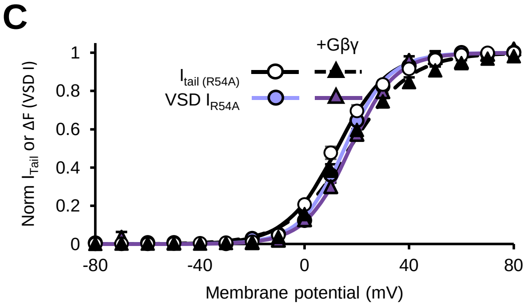

D

Table: R54A, VSD I

	Pore	Pore + Gβγ	VSDI	VSDI + Gβγ
V_{50} (mV)	12±1.4	17±1.8	15±0.4	17±0.7
Z (e^0)	2.7±0.1	2.3±0.2	2.9±0.1	3.0±0.2
n	3	5	3	5

Fig. S3: R54A prevents Gβγ inhibition of Cav2.2-VSD I. (A) Left: voltage-dependent activation of VSD I, right: pre-pulse facilitation **(B)** as in A, but in the presence of Gβγ. **(C)** Voltage-dependent channel opening (I_{tail}) and fluorescence deflections (ΔF) from VSD I. **(D)** Summary of voltage-dependence properties.

Movie S1: Structural dynamics of Cav2.2 during activation by a DRG action potential. Distinct functional domains of the human Cav2.2 channel structure (PDB ID:7MIY) (*6*) were rendered so that their activities are shown as changes in brightness. The data were collected using VCF with action-potential clamp, using the DRG action-potential waveform, in the absence of G-protein regulation (Fig.5A, left panels). Pore opening (conductance) is shown as purple illumination, while the activations of VSDs I, III and IV are shown in blue, green and orange, respectively. VSD II is shown as having constant 0 brightness (no activation), as expected from its apparent lack of voltage sensitivity (Figs.2, S1, S2).
